# Diagnostic utility and psychometric properties of the Technology Activities of Daily Living Questionnaire (T-ADLQ) in people with non-formally educated with Alzheimer’s disease in Lima, Peru

**DOI:** 10.3389/fnagi.2025.1660345

**Published:** 2025-11-12

**Authors:** Rosa Montesinos, Loreto Olavarria, Fernando Henriquez, Diego Chambergo-Michilot, Belén Custodio, Carolina Delgado, Marco Malaga, Nilton Custodio, Andrea Slachevsky

**Affiliations:** 1 Instituto Peruano de Neurociencias, Lima, Peru; 2 Centro de Investigación del Envejecimiento, Facultad de Medicina, Universidad de San Martín de Porres, Lima, Peru; 3 Unidad de Investigación, Equilibria, Lima, Peru; 4 Geroscience Center for Brain Health and Metabolism (GERO), Santiago, Chile; 5 Neuropsychology and Clinical Neuroscience Laboratory (LANNEC), Physiopathology Department – Institute of Biomedical Sciences (ICBM), Neuroscience and East Neuroscience Departments, Faculty of Medicine, University of Chile, Santiago, Chile; 6 Escuela de Psicología, Facultad de Medicina, Universidad Mayor, Santiago, Chile; 7 Laboratory for Cognitive and Evolutionary Neuroscience (LaNCE), Department of Psychiatry, Faculty of Medicine, Pontificia Universidad Católica de Chile, Santiago, Chile; 8 Facultad de Medicina Humana, Universidad Científica del Sur, Lima, Peru; 9 Department of Neurology and Neurosurgery, Clinical Hospital of the University of Chile, Santiago, Chile; 10 Memory and Neuropsychiatric Center (CMYN) Neurology Department, Hospital del Salvador and Faculty of Medicine, University of Chile, Santiago, Chile; 11 Servicio de Neurología, Departamento de Medicina, Clínica Alemana, Universidad del Desarrollo, Santiago, Chile

**Keywords:** activities of daily living, functional assessment and evaluation, informant reported questionnaire, dementia, Alzheimer’s disease, psychometric properties, validation study, Peru

## Abstract

**Objective:**

To evaluate the discriminative capacity and psychometric properties of the Technology–Activities of Daily Living Questionnaire (T-ADLQ) in distinguishing cognitively unimpaired individuals from those with amnestic mild cognitive impairment (aMCI) and Alzheimer’s disease dementia (ADD) in a population with no formal education.

**Methods:**

This cross-sectional study included individuals with no formal education over 50 years of age residing in Callao, Peru. Participants were classified into three cognitive groups: cognitively unimpaired (CU; *n* = 64), aMCI (*n* = 60), and early ADD (*n* = 63). Functional decline was assessed with the T-ADLQ. Group comparisons were conducted using the chi-square or ANOVA tests, as appropriate. Pearson correlations were used to assess concurrent validity. The reliability of the T-ADLQ was assessed using Cronbach’s alpha. Receiver operating characteristic (ROC) curve analyses and area under the curve (AUC) metrics were used to assess the discriminative validity of the measures across the three cognitive groups.

**Results:**

In a sample of 187 illiterate older adults, the T-ADLQ demonstrated excellent internal consistency (Cronbach’s α = 0.966) and strong inverse correlations with global and executive cognitive measures (MMSE, RUDAS, IFS). It also showed a moderate positive correlation with PFAQ. ROC analyses revealed excellent discriminative performance of the T-ADLQ. The total score and the instrumental (IADL) and advanced (AADL) domains achieved perfect accuracy in differentiating cognitively unimpaired individuals from those with aMCI or ADD. The basic activities of daily living (BADL) domain also showed high accuracy, particularly in distinguishing aMCI from ADD.

**Conclusion:**

The T-ADLQ and its subdomains exhibit strong psychometric properties and high discriminative capacity in detecting functional decline in individuals with aMCI and ADD. These findings support the T-ADLQ as a valid and reliable tool for assessing functional impairment in populations with no formal education.

## 1 Introduction

The prevalence of dementia has increased in recent years and is projected to reach 78 million cases by 2030 (Shin, 2022). In addition, it is associated with a higher risk of mortality (Wetterberg et al., 2022) and a progressive decline in quality-of-life (Van De Beek et al., 2019), making it a significant public health concern. There are notable disparities in the incidence of dementia, with most cases projected to increase in low- and middle-income countries (LMIC) where large segments of the population have limited formal education–a known risk factor for dementia, as shown by international analyses linking lower educational attainment to increased dementia risk among Caribbean populations as well as Hispanic, Black, and White Americans in the United States (Li et al., 2021; Nichols et al., 2022). Furthermore, underdiagnosis is more prevalent among individuals with limited educational backgrounds compared to those with higher education (Amjad et al., 2018). One of the main barriers to diagnosis in these populations is the lack of assessment tools specifically designed for individuals with limited formal education, as most screening instruments exhibit substantial educational bias (Parra et al., 2021).

The diagnosis of dementia is based on an acquired decline in cognitive capacity that leads to functional impairment, interfering with the ability to remain independent in everyday activities (Sachdev et al., 2015). Therefore, identifying impairment in the capacity to perform activities of daily life (ADL) secondary to cognitive decline is a cornerstone in the diagnosis of dementia (Slachevsky et al., 2024). Although several tools exist for assessing ADL in LMICs and the global south, most have been validated without accounting for participants’ educational level (Iavarone et al., 2007; Custodio et al., 2020, 2022; Rodríguez Félix, 2021; Pellicer-Espinosa and Díaz-Orueta, 2022).

Activities of Daily Livings are commonly classified according to their cognitive complexity, ranging from routine to more demanding tasks. Within this framework, ADLs are typically divided into three categories: basic (BADLs), instrumental (IADLs), and advanced (AADLs) (Slachevsky et al., 2024). The specific activities encompassed by IADLs and AADLs may vary depending on individual characteristics–such as educational level and gender–as well as broader sociocultural factors (Yemm et al., 2021; Nichols et al., 2023). For example, the growing integration of information and communication technologies into daily life has transformed the nature and demands of certain IADLs and AADLs tasks (Slachevsky et al., 2024).

While the initial operational criteria for mild cognitive impairment (MCI) excluded functional impairment (Morris, 2012), growing evidence indicates that individuals with MCI may exhibit subtle deficits in complex ADLs (Sachdev et al., 2014). Reflecting this, the DSM-5 criteria for MCI allow for mild functional impairment, provided it does not compromise overall independence.

Despite significant progress in developing and validating cognitive assessment tools for individuals with little or no formal education (Custodio et al., 2019, 2020), the evaluation of functional abilities–particularly ADLs–has received comparatively less attention (Yemm et al., 2021; Nichols et al., 2023; Slachevsky et al., 2024). Few studies have explored the clinical utility of ADL assessments in these populations. This gap is particularly pronounced in Latin America, where culturally and educationally appropriate tools for evaluating ADLs in low-educated individuals remain scarce (Slachevsky et al., 2024).

Given the high prevalence of low educational attainment in many LMIC, such as among Latin American older adults, there is a pressing need to develop and validate instruments capable of assessing functional impairment across the full range of ADL complexity in populations without formal education in these populations.

Although older adults in Latin America with no formal education represent a sizable population, they remain underrepresented in research (Ciocca and Delgado, 2017; Zacca-González et al., 2014). Only three studies have examined functional ability in this group. Sposito et al. (2016) found that cognitively unimpaired individuals without schooling engaged less in social, intellectual, and physical ADLs than those with education. Brigola et al. (2019) reported poorer IADL performance among older adults with no formal education, while data from the Mexican Health and Aging Study showed a 0.06-point reduction in Katz BADL scores per additional year of schooling (Díaz-Venegas and Wong, 2016). These findings suggest that activity limitations may inflate MCI or dementia diagnoses, yet this possibility has not been directly tested. Prior studies excluded participants with cognitive impairment and focused narrowly on selected BADL or IADL, overlooking advanced and technology-related activities–a gap that the present study addresses.

This issue is particularly relevant for three reasons. First, as outlined above, education has been shown to influence the types of ADL performed, particularly IADL and AADL. Second, in Alzheimer’s disease (AD), a hierarchical pattern of functional decline has been consistently documented, with impairments in AADL and IADL emerging prior to deficits in BADL (Mioshi et al., 2007; Giebel et al., 2020). Third, functional assessments in individuals with limited or no formal education have been shown to be less susceptible to educational bias than cognitive tests (Moon et al., 2024), thereby providing valuable diagnostic information in the evaluation of dementia (Mograbi et al., 2014; González et al., 2021; Kosmidis, 2018).

The Technology–Activities of Daily Living Questionnaire (T-ADLQ) is an informant-rated tool designed to assess a broad spectrum of ADL, including the use of information and communication technologies (ICTs), across varying levels of complexity: BADLs, IADLs, and AADLs. Previous studies have demonstrated its strong clinical utility and reliability in detecting functional impairment associated with aging and diverse neurocognitive disorders (Muñoz-Neira et al., 2012; Idiáquez et al., 2017; Delgado et al., 2019; Musa Salech et al., 2022). However, its diagnostic performance in populations with no formal education remains unexamined.

To address this gap, it is essential to understand patterns of technology access and use among individuals with low educational attainment, as these factors may influence the applicability of tools like the T-ADLQ. In Peru, internet penetration increased steadily between 2013 and 2022, although notable disparities persist between fixed and mobile services. While fixed internet access reached less than 10% of the population, mobile internet usage expanded rapidly, reaching nearly 90% of the population by 2022 (Gamboa and García, 2024). In Peru, ICT penetration has expanded considerably in recent years, even among households headed by individuals with only primary or lower levels of education. For instance, by early 2024, 88.4% of these households had access to at least one ICT resource, such as a mobile phone, television with cable, computer, or internet connection (Instituto Nacional de Estadística e Informática [INEI], 2024). Similarly, earlier reports indicated that over 87% of these households had access to mobile phones (Calderón, 2021). These trends suggest a growing integration of technology into daily life, supporting the applicability of ICT-based functional assessments like the T-ADLQ, even in low-literacy contexts.

The objective of this study was to evaluate the discriminative capacity and psychometric performance of the T-ADLQ in differentiating individuals with Alzheimer’s disease dementia (ADD) and amnestic mild cognitive impairment (aMCI) from cognitively unimpaired (CU) individuals in a population lacking formal education.

## 2 Materials and methods

### 2.1 Study design and population

This cross-sectional observational study was conducted in collaboration with the Regional Health Directorate of Callao (DIRESA Callao), located in the coastal region adjacent to Lima, Peru. The study targeted adults aged 50 and older with no formal education, residing in the urban district of Ventanilla, Callao province. Participants were recruited through the Pachacútec healthcare network, which encompasses several public health centers, including Perú-Corea Pachacútec, 03 de Febrero, Santa Rosa de Pachacútec, Bahía Blanca, and Ciudad Pachacútec. Further details on the healthcare setting and recruitment process are available in Montesinos et al. (2022).

Participants were categorized into three groups: CU, aMCI and early ADD. No formal education was defined as less than 1 year of schooling and an inability to read or write.

Exclusion criteria included significant sensory or motor impairments affecting test performance; non-Spanish speakers; functionally literate individuals (fewer than 4 years of schooling before age 15 but able to read, write, and function socially); moderate-to-severe dementia; cerebrovascular disease; developmental or intellectual disabilities; history of traumatic brain injury; psychiatric disorders (including depression, psychosis, bipolar disorder, schizophrenia, or personality disorders); and substance use disorders. Depression was screened using the Depression Inventory-II (BDI-II) (Vega-Dienstmaier et al., 2014).

### 2.2 Ethical considerations

The study protocol was approved by the Ethics Committee and Postgraduate Section of the Faculty of Human Medicine, Universidad de San Martín de Porres (Lima, Peru). Authorization to conduct the study in participating healthcare centers was granted by DIRESA Callao. Informed consent was obtained from all participants, either by signature or fingerprint, using approved consent forms, in accordance with the Declaration of Helsinki.

### 2.3 Study procedures

Following institutional approval, the research team coordinated with nursing staff at each health center to introduce the study objectives and foster collaboration. Prior to data collection, a standardized training program was implemented.

Training was provided to psychology students, primary care physicians, geriatric medicine residents, and neuropsychologists affiliated with the Diagnosis of Cognitive Impairment and Dementia Prevention Unit (UDDCPD) at the Peruvian Institute of Neurosciences (IPN). It covered the study protocol and administration of cognitive and functional assessments: Mini-Mental State Examination (MMSE) (Custodio and Lira, 2014), Rowland Universal Dementia Assessment Scale – Peruvian version (RUDAS-PE) (Custodio et al., 2019), INECO Frontal Screening (IFS) (Custodio et al., 2016), Pfeffer Functional Activities Questionnaire (PFAQ), and the T-ADLQ. Neuropsychologists also received instruction in applying diagnostic criteria for aMCI and early ADD.

### 2.4 Clinical evaluation

Assessments were conducted during scheduled visits to participating health centers. A trained interviewer administered a structured demographic and health questionnaire, conducted a brief neurological exam, and recorded anthropometric data and blood pressure. Information on comorbidities and current medications (as of the day before assessment) was also collected.

### 2.5 Diagnostic criteria for aMCI and early ADD

Diagnostic classification followed by a three-phase process: screening, clinical evaluation, and final classification.

Phase 1: Screening

In Phase 1, conducted in primary care centers in Ventanilla (Callao, Peru), participants were evaluated by trained field assessors (medical and psychology students and interns). All participants underwent cognitive screening with the MMSE and a brief functional assessment using the PFAQ, based on which they were preliminarily classified into three groups:

- Group 1: MMSE < 18 and PFAQ ≥ 7 = dementia
- Group 2: MMSE < 18 and PFAQ < 6 = MCI
- Group 3: MMSE > 18 and PFAQ < 6 = CU.

Phase 2: Clinical Evaluation

Phase 2 was conducted in primary care centers in Ventanilla (Callao, Peru) by a different team of professionals from those in Phase 1. This team included neurologists and geriatricians affiliated with the UDDCPD-IPN, as well as neuropsychologists and neuro-rehabilitation specialists from the IPN.

Participants referred from Phase 1 underwent a comprehensive neuropsychological assessment using the Uniform Data Set Version 3 Neuropsychological Battery (UDS3-NB), specifically adapted for individuals with low levels of education or no formal education (Besser et al., 2018). This battery included assessments of multiple cognitive domains: Attention (Number Span Test); Processing speed (Trail Making Test Part A); Executive functioning (Trail Making Test Part B); Naming (Multilingual Naming Test, MINT); Phonemic fluency (Letters P and M); Semantic fluency (Animal and Vegetable Naming); Visuospatial skills (Benson Complex Figure; Copy Learning and memory (Craft Story 21, CS-21); Immediate and Delayed Recall (BCF – Recall).

Cognitive impairment was defined as a score of two or more standard deviations below the normative mean in each domain.

The Clinical Dementia Rating (CDR) was administered during a joint interview with both the participant and their caregiver or companion and was scored independently by two blinded evaluators (a neuropsychologist and a neuro-rehabilitation specialist from IPN) (Morris et al., 1997).

Any discrepancies in classification were resolved through multidisciplinary consensus involving neurologists, geriatricians, neuropsychologists, and neuro-rehabilitation specialists at IPN.

Importantly, T-ADLQ results were not used for diagnostic purposes and were blinded to the evaluators throughout the classification process.

Finally, the three groups were referred to the UDDCPD-IPN for re-evaluation in a third diagnostic phase, during which the diagnoses of aMCI and ADD were confirmed.

Phase 3: Diagnostic Classification

In Phase 3, final diagnostic classification was refined by incorporating additional clinical information, including laboratory test results and neuroimaging findings. All participants underwent a standard panel of laboratory tests–complete blood count (CBC), fasting glucose, creatinine, liver function tests, vitamin B12, thyroid hormone levels, rapid plasma reagin (RPR), and HIV-ELISA–to rule out reversible or secondary causes of cognitive impairment, such as vitamin B12 deficiency, hypothyroidism, or infectious etiologies. In parallel, a non-contrast computed tomography (CT) scan of the brain was performed to evaluate structural abnormalities and to exclude differential diagnoses such as normal pressure hydrocephalus, tumors, or significant cerebrovascular disease.

The final diagnostic subtypes–aMCI or ADD–were established according to DSM-5 and NIA-AA criteria. Diagnoses were established through a comprehensive integration of CDR scores, neuropsychological test performance (see Phase 2), biochemical markers, and brain imaging findings (Phase 3). Only participants who met all DSM-5 and NIA-AA criteria for aMCI or ADD across these domains were included in the study groups. CU participants, initially identified during Phase 1 based on non-pathological performance on the MMSE and PFAQ, also underwent a full re-evaluation during this phase. This included neuropsychological testing, laboratory analyses, and brain imaging to ensure accurate classification and confirm the absence of cognitive impairment or underlying pathology.

Final Sample

Participants were classified into three groups: three groups: CU (MMSE > 18; PFAQ < 6 and CDR = 0), aMCI ((MMSE > 18; PFAQ < 6 and CDR = 0.5), defined according to the NIA-AA criteria for aMCI and ADD (MMSE < 18; PFAQ > 7 and CDR = 1 or 2), defined according to the National Institute on Aging-Alzheimer’s Association (NIA-AA) criteria for early-stage ADD (Jack et al., 2018, 2024). The final sample included 64 CU, 60 individuals with aMCI, and 63 with early ADD.

### 2.6 Measures

#### 2.6.1 Technology-activities of daily living questionnaire (T-ADQL)

The T-ADLQ is an informant-based instrument developed to assess functional abilities by evaluating performance across a range of ADLs. It is completed by a reliable informant–typically a family member or primary caregiver–particularly when evaluating individuals with dementia. The T-ADLQ comprises 33 items categorized into three domains based on the complexity of the tasks: BADLs, IADLs, and AADLs (Delgado et al., 2019; Slachevsky et al., 2019). These are further subdivided into seven functional subscales: Self-Care, Household Care, Employment and Recreation, Shopping and Money Management, Travel, Communication, and Technology Use.

Each item is rated on a 4-point Likert scale, ranging from 0 (no difficulty) to 3 (unable to perform the activity). In addition, two non-numerical response options are included: “Never Did” (ND), for activities not performed prior to dementia onset (e.g., employment), and “Don’t Know” (DK), for situations in which the informant lacks sufficient information. These options allow for adjustment based on premorbid functioning and help mitigate potential cultural or gender-related biases.

The global and subscale scores are calculated using the formula: Total Score (excluding ND and DK responses) ÷ [3× number of items answered (excluding ND and DK)]. This approach ensures that only applicable and known activities are included in the final score, providing a more accurate representation of current functional impairment relative to premorbid capacity.

Scores are expressed as percentages, with higher values indicating greater levels of functional impairment (range: 0%–100%). Both domain-specific and global indices of functional decline can be derived from the instrument.

#### 2.6.2 Brief neuropsychological tests and other functional measures

Neuropsychological measures included three previously validated instruments that have demonstrated adequate internal consistency, sensitivity, and specificity: the RUDAS-PE (total score = 30), the MMSE (total score = 30), and the IFS (total score = 30).

Concurrent validity of the T-ADLQ was evaluated with the PFAQ, an informant-based measure of IADLs, completed by the same proxy informant who responded to the T-ADLQ. It consists of 10 items covering domains such as managing finances, handling medications, and shopping. Each item is rated on a 4-point scale: 0 = normal or never did but could do now; 1 = has difficulty but does independently or never did but would have difficulty now; 2 = require assistance; and 3 = dependent. Total scores range from 0 to 30, with higher scores reflecting greater functional impairment. Notably, the PFAQ does not assess BADLs or more complex activities such as social participation and employment. Moreover, it lacks an option to indicate whether an individual has never performed a given activity, which limits its capacity to adjust for sociodemographic background and lifetime exposure to certain tasks.

### 2.7 Statistical analysis

Categorical variables were described as frequencies and percentages, and their comparisons across the three groups (CU, aMCI, and ADD) were conducted using the Chi-square (χ^2^) test. Continuous variables were reported as means with standard deviations and compared across groups using one-way analysis of variance (ANOVA) followed by Bonferroni *post hoc* correction to adjust for multiple comparisons. Effect sizes were reported using partial eta squared (η^2^_*P*_), interpreted according to Cohen’s guidelines (0.01–0.05 as small, 0.06–0.13 as medium, and ≥0.14 as large) (Sullivan and Feinn, 2012).

The internal consistency of the T-ADLQ was evaluated using Cronbach’s alpha, reflecting the average inter-item correlation. Concurrent validity was assessed through Pearson correlation coefficients between T-ADLQ scores and both functional (PFAQ) and cognitive measures (MMSE, RUDAS, and IFS).

To determine discriminative capacity, Receiver Operating Characteristic (ROC) curve analyses were performed to assess the ability of the T-ADLQ to distinguish among the three diagnostic groups: (1) CU vs. ADD, (2) CU vs. aMCI, and (3) aMCI vs. ADD. The optimal cut-off scores for each comparison were derived from the area under the curve (AUC). Sensitivity and specificity were calculated for the total T-ADLQ score as well as for its subdomains: BADL, IADL, and AADL.

Analysis was conducted using the Statistical Package for the Social Sciences (SPSS) version 22 for Windows (IBM Corp., Armonk, NY, USA). A *p*-value of <0.05 was considered indicative of statistical significance.

## 3 Results

### 3.1 Demographic and clinical data

The total sample consisted of 187 non-formal education older adults, distributed across three diagnostic groups: 64 CU (36 women), 60 individuals with aMCI (34 women), and 63 patients with ADD (35 women). Table 1 presents the demographic and clinical characteristics of the sample.

**TABLE 1:** Demographic, neuropsychological, and T-ADLQ data.

	CU	aMCI	ADD	*P* (global)	*P* (*Post-hoc*)
Number of cases	64	60	63		
Male/female	28 (44%)/36 (56%)	26 (43%)/34 (57%)	28 (44%)/35 (56%)	0.992^a^	–
Age	68.92 ± 3.45 (60–82)	68.77 ± 3.14 (62–76)	72.70 ± 3.42 (67–81)	**<0.001^b^**	P1 < 0.001 P2 = 1.000 P3 < 0.001
MMSE (total score: 30)	20.16 ± 1.49 (17–23)	17.85 ± 1.65 (14–22)	10.11 ± 1.58 (7–14)	**<0.001^b^**	P1 < 0.001 P2 < 0.001 P3 < 0.001
RUDAS (total score: 30)	23.88 ± 0.93 (22–25)	20.43 ± 1.39 (16–23)	14.97 ± 2.21 (10–20)	**<0.001^b^**	P1 < 0.001 P2 < 0.001 P3 < 0.001
IFS (total score: 30)	24.06 ± 1.11 (22–27)	19.90 ± 1.34 (17–23)	14.25 ± 1.96 (10–18)	**<0.001^b^**	P1 < 0.001 P2 < 0.001 P3 < 0.001
BDI-II (total score: 63	5.94 ± 2.82 (0–12)	6.20 ± 2.94 (0–12)	7.23 ± 3.106 (0–12)	**<0.034^b^**	P1 = 0.041 P2 = 1.000 P3 = 0.156
PFAQ (total score 33)	2.23 ± 1.32 (0–5)	3.73 ± 2.10 (0–11)	16.56 ± 4.84 (11–26)	**<0.001^b^**	P1 < 0.001 P2 = 0.026 P3 < 0.001
Total scale T-ADLQ (0%–100%)	9.28 ± 5.87 (0–25.64)	45.04 ± 5.35 (34.44–56.25)	51.72 ± 12.75 (36.67–84.52)	**<0.001^b^**	P1 < 0.001 P2 < 0.001 P3 < 0.001
Basic T-ADLQ (BADL) (0%–100%)	3.33 ± 3.94 (0–20.00)	10.67 ± 4.28 (0–20.00)	32.70 ± 20.97 (13.33–80.00)	**<0.001^b^**	P1 < 0.001 P2 = 0.004 P3 < 0.001
Instrumental T-ADLQ (IADL) (0%–100%)	10.93 ± 7.73 (0–33.33)	53.34 ± 5.80 (41.27–66.67)	55.68 ± 12.48 (35.00–91.23)	**<0.001^b^**	P1 < 0.001 P2 < 0.001 P3 = 0.474
Advanced T-ADLQ (AADL) (0%–100%)	8.39 ± 7.29 (0–33.33)	46.51 ± 11.58 (16.67–71.43)	54.80 ± 13.89 (33.33–100)	**<0.001^b^**	P1 < 0.001 P2 < 0.001 P3 < 0.001

Data are presented in mean ± standard deviation (minimum–maximum);

a, Chi-square;

b, One-way ANOVA (*Post-hoc*: Bonferroni test); CU, cognitively unimpaired; aMCI, amnestic mild cognitive impairment; ADD, Alzheimer’s disease dementia; MMSE, Mini-Mental State Examination; RUDAS, Rowland Universal Dementia Assessment Scale; IFS, INECO Frontal Screening; BDI-II, Beck Depression Inventory-II; PFAQ, Pfeffer Functional Activities Questionnaire; T-ADLQ, the Technology Activities of Daily Living Questionnaire; BADL, basic activities of daily living; IADL, Instrumental Activities of Daily Living; AADL, Advanced Activities of Daily Living; P1, values refer to the comparison between CU group and AD; P2, values refer to the comparison between CU group and MCI; P3, values refer to the comparison between MCI group and AD. *p*-value < 0.05. Bold values indicated significant difference between study groups.

No significant differences were found in sex distribution among the groups [χ^2^ (2) = 0.16, *p* = 0.992]. In contrast, significant group differences were observed in age [*F*(2, 184) = 27.81, *p* < 0.001, η^2^_*P*_ = 0.23]. *Post hoc* analyses using Bonferroni correction indicated that the group of patients with ADD differed significantly from both CU and individuals with aMCI. No significant age differences were observed between the CU and the aMCI groups.

Significant differences were also found across cognitive and functional measures, including the MMSE [*F*(2, 184) = 707.86, *p* < 0.001, η^2^_*P*_ = 0.89], RUDAS [*F*(2, 184) = 496.73, *p* < 0.001, η^2^_*P*_ = 0.84], IFS [*F*(2, 184) = 671.73, *p* < 0.001, η^2^_*P*_ = 0.88], and PFAQ [*F*(2, 184) = 394.13, *p* < 0.001, η^2^_*P*_ = 0.81]. *Post hoc* analyses using Bonferroni correction revealed that individuals with ADD performed significantly worse than both the CU and aMCI groups on all measures. Comparisons between CU and aMCI also showed significant differences in cognitive and functional performance.

### 3.2 Performances in the technology-activities of daily living questionnaire (T-ADLQ)

**FIGURE 1:**
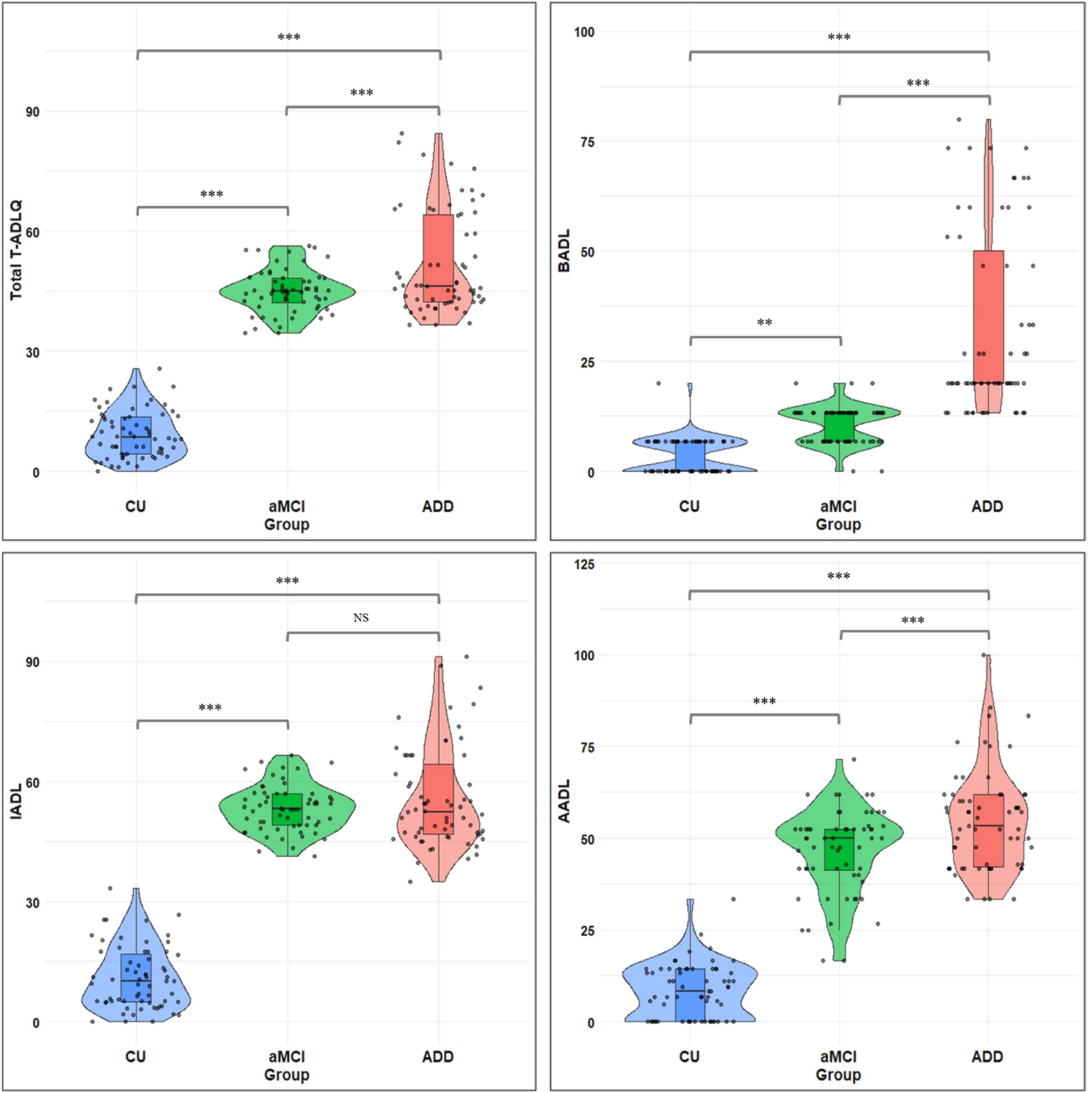
Total and subdomain T-ADLQ performance across CU, aMCI, and ADD groups. T-ADLQ, the Technology Activities of Daily Living Questionnaire; BADL, basic activities of daily living; IADL, Instrumental Activities of Daily Living; AADL, Advanced Activities of Daily Living; CU, Cognitively Unimpaired; aMCI, amnestic mild cognitive impairment; ADD, Alzheimer’s Disease Dementia; NS, non-significant differences; ***p < 0.001; **p < 0.01.

Regarding T-ADLQ performance, significant group differences were found in the total score [*F*(2, 184) = 435.60, *p* < 0.001, η^2^_*P*_ = 0.83], as well as in all three domains: BADL [*F*(2, 184) = 92.56, *p* < 0.001, η^2^_*P*_ = 0.50], IADL [*F*(2, 184) = 479.03, *p* < 0.001, η^2^_*P*_ = 0.84], and AADL [*F*(2, 184) = 307.58, *p* < 0.001, η^2^_*P*_ = 0.77]. *Post hoc* analyses revealed significant differences among the three groups in the total T-ADLQ score, as well as in BADL and AADL. For IADL, differences were observed between CU and aMCI, and between CU and ADD, but not between aMCI and ADD (Figure 1 and Table 1).

### 3.3 Internal consistency

The internal consistency of the 33 items comprising the T-ADLQ was excellent (Cronbach’s α-coefficients = 0.966). At the domain level, internal consistency was moderate for BADL (Cronbach’s α-coefficients = 0.654), and good for IADL (Cronbach’s α-coefficients = 0.874) and AADL (Cronbach’s α-coefficients = 0.865).

### 3.4 Concurrent validities

Regarding concurrent validity, the global T-ADLQ score demonstrated a strong positive correlation with the PFAQ (Pearson’s *r* = 0.657, *p* < 0.001), and strong negative correlations with the MMSE (*r* = −0.682, *p* < 0.001), the RUDAS-PE (*r* = −0.758, *p* < 0.001), and the IFS (*r* = −0.779, *p* < 0.001) (Table 2). The three T-ADLQ subdomains–BADL, IADL), and a-ADL–also showed adequate concurrent validity, with significant correlations observed with both the PFAQ and cognitive screening tests (see Table 2 for details).

**TABLE 2:** Concurrent validity of T-ADLQ.

	Instruments	Total T-ADLQ		BADL		IADL		AADL	
		*r*	*p*	*r*	*p*	*r*	*p*	*r*	*p*
**Global cognition**									
	MMSE	−0.682	<0.001	−0.689	<0.001	−0.631	<0.001	−0.659	<0.001
	RUDAS	−0.758	<0.001	−0.700	<0.001	−0.715	<0.001	−0.731	<0.001
**Executive function**									
	IFS	−0.779	<0.001	−0.693	<0.001	−0.740	<0.001	−0.754	<0.001
**Functional capacity**									
	PFAQ	0.675	<0.001	0.843	<0.001	0.597	<0.001	0.633	<0.001

T-ADLQ, the Technology Activities of Daily Living Questionnaire; MMSE, Mini-Mental State Examination; RUDAS, Rowland Universal Dementia Assessment Scale; IFS, INECO Frontal Screening; PFAQ, Pfeffer Functional Activities Questionnaire.

### 3.5 Discriminative capacities

**FIGURE 2:**
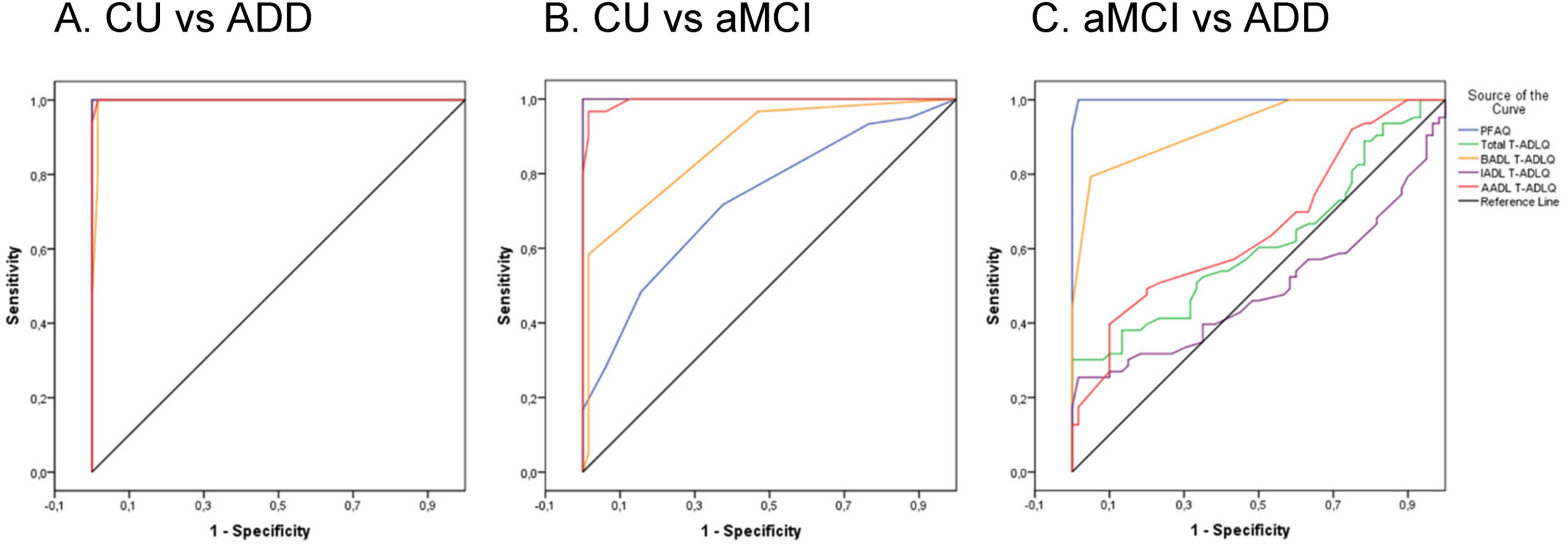
Receiver operating characteristic (ROC) curves for the total T-ADLQ, BADL, IADL, AADL, and PFAQ in discriminating between diagnostic groups. **(A)** CU vs. ADD. **(B)** CU vs. aMCI. **(C)** aMCI vs. ADD. CU, Cognitively unimpaired; ADD, Alzheimer’s Disease Dementia; aMCI, amnestic mild cognitive impairment.

The results of the ROC curve analysis for the T-ADLQ and its domains are summarized in Table 3, including AUC values, cut-off points, sensitivity, and specificity. Although the PFAQ was used as part of the diagnostic criteria during participant inclusion, its results are also presented in the table for comparative purposes with the T-ADLQ. For distinguishing CU from individuals with ADD (Figure 2A), the total T-ADLQ, IADL, and AADL domains showed perfect discriminative ability (AUC = 1.000), while the BADL domain also performed excellently (AUC = 0.994). In the CU vs. aMCI comparison (Figure 2B), the total T-ADLQ and IADL domains again reached an AUC of 1.000, followed by AADL (AUC = 0.995) and BADL (AUC = 0.874). For discriminating between aMCI and ADD (Figure 2C), only the BADL domain showed good performance (AUC = 0.926), whereas total T-ADLQ, IADL, and AADL domains showed limited discriminative capacity.

**TABLE 3:** Receiver operating characteristic (ROC) analysis of T-ADLQ and its three subdomains in cognitively unimpaired, amnesic MCI, and Alzheimer’s disease dementia individuals.

Domain	Group comparison	AUC	Cut-off	Sensitivity (%)	Specificity (%)
Total T-ADLQ	CU vs. ADD	1.000	31.15	100.0	100.0
	CU vs. aMCI	1.000	30.04	100.0	100.0
	aMCI vs. ADD	0.612	45.20	57.1	53.3
IADL	CU vs. ADD	1.000	34.16	100.0	100.0
	CU vs. aMCI	1.000	37.30	100.0	100.0
	aMCI vs. ADD	0.491	51.76	52.4	41.7
AADL	CU vs. ADD	1.000	28.57	100.0	98.4
	CU vs. aMCI	0.995	24.40	96.7	98.4
	aMCI vs. ADD	0.656	51.19	57.1	56.7
BADL	CU vs. ADD	0.994	10.00	100.0	98.4
	CU vs. aMCI	0.874	3.33	96.7	53.1
	aMCI vs. ADD	0.926	16.66	79.4	95.0
PFAQ	CU vs. ADD	1.000	8.00	100.0	100.0
	CU vs. aMCI	0.728	2.50	71.7	62.5
	aMCI vs. ADD	0.999	10.50	100.0	98.3

T-ADLQ, the Technology Activities of Daily Living Questionnaire; BADL, basic activities of daily life; IADL, Instrumental Activities of Daily Life; AADL, Advanced Activities of Daily Living; PFAQ, Pfeffer Functional Activities Questionnaire; CU, cognitively unimpaired; aMCI, amnestic mild cognitive impairment; ADD, Alzheimer’s disease dementia.

## 4 Discussion

This study aimed to validate the T-ADLQ and evaluate its discriminative capacity in older adults with no formal education diagnosed with aMCI or ADD. To our knowledge, few studies have explored functional impairment across varying levels of ADL complexity, including traditional and technology-based instrumental and advanced activities. Our results indicate that T-ADLQ, along with its three subdomains, demonstrates adequate convergent validity and internal consistency. Notably, both the total T-ADLQ score and the BADL subdomain exhibit strong diagnostic accuracy in distinguishing CU individuals from those with aMCI and ADD. In contrast, the AADL and IADL subdomains show limited ability to differentiate between aMCI and ADD.

Our findings are also consistent with those of Idiáquez et al. (2017), who demonstrated the utility of the T-ADLQ in detecting functional impairments in individuals with minor stroke. Although 13% of their participants had between 0 and 4 years of formal education, the study did not examine how educational level might influence T-ADLQ performance, leaving this important factor unaddressed. Moreover, other validation studies of the T-ADLQ have typically excluded illiterate populations, highlighting the novelty and relevance of our study in this underserved group (Muñoz-Neira et al., 2012; Delgado et al., 2019; Musa Salech et al., 2022).

The total T-ADLQ score showed perfect discriminative capacity in distinguishing CU individuals from those with ADD, achieving an AUC of 1.000 in the ROC analysis. An optimal cut-off of 31.15 yielded 100% sensitivity and specificity. This threshold is slightly higher than that reported by Muñoz-Neira et al. (2012), with a modest absolute difference of 1.9 points on a 100-point scale. However, this variation may reflect differences in sample characteristics, particularly educational level or technological familiarity. In Muñoz-Neira’s study, mean years of schooling ranged from 10.76 to 13.11 across diagnostic groups (CU, MCI, or dementia of different etiology). Given that performance on several IADLs and AADLs are influenced by education, individuals with limited or no formal schooling may score higher on the T-ADLQ despite cognitive impairment. As a result, a higher cut-off may be necessary to accurately distinguish CU individuals from those with ADD in populations with low education levels. These findings highlight the importance of adjusting diagnostic thresholds to the sociocultural and functional context of the population to enhance diagnostic precision and reduce misclassification. Nonetheless, the clinical relevance of these differences should be further explored in similarly characterized cohorts.

The IADL and AADL domains also achieved perfect discriminative capacity (AUC = 1.000) in distinguishing CU individuals from those with ADD. The BADL domain also demonstrated excellent performance, with a high AUC of 0.994. These findings are consistent with previous literature indicating that functional impairments become more pronounced and pervasive as Alzheimer’s disease advances (Cornelis et al., 2019). In moderate stages of ADD, difficulties typically emerge not only in AADL and IADL, but also in BADL, potentially resulting in consistent deficits across multiple domains assessed by the T-ADLQ. For the CU vs. aMCI contrast, the total T-ADLQ and IADL domains again yielded AUC values of 1.000, with the AADL and BADL domains showing slightly lower values (AUC = 0.995 and 0.874, respectively). Notably, the T-ADLQ demonstrated superior discriminative capacity compared to the PFAQ in differentiating CU individuals from those with aMCI (AUC = 1.000 vs. 0.728). The T-ADLQ may be more sensitive to early functional changes characteristic of the prodromal stages of AD, as it includes AADL rather than being limited to IADL like the PFAQ. Our findings align with current evidence supporting a continuum of functional impairment across the AD spectrum, beginning as early as the MCI stage, particularly in complex ADL (Raimo et al., 2024). Consistent with our findings, Montesinos et al. (2025) recently reported results from a memory clinic in Lima involving individuals with middle and higher levels of education. In their study, the Amsterdam Instrumental Activities of Daily Living Questionnaire–short version (A-IADL-Q-SV) demonstrated excellent accuracy in classifying dementia severity and showed strong correlations with cognitive and other functional measures. Their sample included 171 participants (81 with CDR = 0, 70 with CDR = 1, and 20 with CDR = 2), among whom 54 had ADD and 36 had frontotemporal dementia (Montesinos et al., 2025).

The higher AUCs observed for the AADL and IADL domains compared to BADL are consistent with previous research indicating that individuals with aMCI generally maintain independence in basic functions but may exhibit subtle impairments in more complex tasks–such as planning, financial management, or social engagement–captured by the IADL and AADL domains (Atri et al., 2025). Despite this general pattern, we unexpectedly observed early impairment in BADL among individuals with aMCI, even though these functions are typically preserved until later stages of AD. One possible explanation is that specific structural or population-level factors–such as low educational attainment, high comorbidity burden, or socioeconomic vulnerability–may contribute to an accelerated functional decline in this population. However, this interpretation remains preliminary and warrants further investigation in cohorts with comparable characteristics (Shin et al., 2015).

From a clinical and public health standpoint, these findings underscore the importance of understanding functional trajectories in populations with low or no formal education. The atypical pattern observed–marked by earlier-than-expected impairment in BADL–may reflect distinct cognitive aging processes influenced by life-course factors such as limited educational attainment, occupational complexity, and reduced cognitive reserve. Recognizing these divergent profiles is critical for developing culturally and contextually appropriate assessment tools, as well as for guiding diagnostic practices and care models that address the specific needs of historically underrepresented populations in dementia research (Sharp and Gatz, 2011; Nitrini et al., 2020).

In comparison between aMCI and ADD, only the BADL subdomain demonstrated high discriminative capacity (AUC = 0.926). In contrast, the total T-ADLQ score, along with the IADL and AADL subdomains demonstrated limited discriminative capacity (AUC = 0.612, 0.491, and 0.656, respectively). This pattern may reflect the gradual and overlapping progression of functional decline from aMCI to ADD during which impairments in IADLs and ADLs become increasingly prevalent in both conditions, thereby limiting their ability to differentiate between these clinical stages (Raimo et al., 2024). However, it is important to acknowledge that using the PFAQ as an inclusion criterion may have introduced recruitment bias by overrepresenting participants already impaired in IADLs. This likely reduced variability in complex ADL–encompassing both IADLs and AADLs–potentially limiting the discriminative power of the corresponding T-ADLQ subscores. Therefore, both the natural overlap between clinical stages and methodological aspects of participant selection may have contributed to the lower discriminative capacity observed in these domains. In addition, IADL and AADL tasks–such as managing money, or IADLs or AADLS completed with ICT–are strongly influenced by education, socioeconomic status, and culture (Dias et al., 2015; Guo and Sapra, 2025). In contrast to IADL and AADL, the BADL subdomain of the T-ADLQ demonstrated a high AUC (0.926), underscoring its potential as a specific marker of progression to established dementia. Remarkably, this discriminative capacity was comparable to that of the PFAQ (AUC = 0.999), despite focusing solely on basic physical self-care abilities. Unlike more cognitively demanding IADLs and AADLs, BADLs are less affected by contextual factors such as educational level or cultural influences–including caregiver expectations and cultural values–which may enhance their diagnostic reliability across diverse populations (Nichols et al., 2023). This is particularly relevant in our study sample of older adults with no formal education, where tasks requiring literacy or numeracy may produce false positive results. In such contexts, the relative robustness of BADL assessments represents a key advantage for accurate functional evaluation. Moreover, because impairments in BADLs tend to emerge more clearly in moderate to severe stages of dementia, their presence could contribute meaningfully to the ability of this subdomain to differentiate between more advanced cases. Therefore, the strong diagnostic performance of the BADL subdomain likely reflects both its resilience to sociodemographic confounders and its heightened sensitivity to significant functional deterioration.

The internal consistency of the T-ADLQ was high (Cronbach’s alpha = 0.966), comparable to the results reported by Muñoz-Neira et al. (2012) in Chile (Cronbach’s alpha = 0.861). Spearman’s correlations between the T-ADLQ, RUDAS, and IFS suggest that the T-ADLQ is a good predictor of both global cognition and executive function. These findings are consistent with previous research highlighting the relationship between executive dysfunction and the loss of instrumental daily living skills, particularly in the progression from MCI to Alzheimer’s Disease (Maeshima et al., 2021). In contrast, the correlation between the T-ADLQ and the PFAQ was moderate (rho = 0.675). This discrepancy may be attributed to the broader scope of the T-ADLQ, which assesses three domains of daily functioning, while the PFAQ focuses exclusively on IADLs. This difference in coverage may influence the sensitivity of each tool in detecting functional changes.

In another aspect, although access to digital technologies has increased in recent years even among individuals with limited formal education, it is important to note that significant digital access barriers persist among older adults in Peru (Barrantes and Ugarte, 2018). Digital illiteracy may affect performance on technology-related items within the T-ADLQ, particularly in AADL items, i.e., internet access, for example (Melrose et al., 2016). Therefore, limited digital literacy should be carefully considered when interpreting functional assessment scores in low-literacy populations, as poor performance in technology-based tasks may reflect reduced access or familiarity with digital tools rather than cognitive impairment *per se*.

Nonetheless, the inclusion of a technological domain in the T-ADLQ (Muñoz-Neira et al., 2012) - encompassing items related to the use of computers, mobile phones, automated teller machines, internet access, and email–may enhance the instrument’s sensitivity for detecting functional decline in contemporary contexts (Slachevsky et al., 2024). It may improve the ability to distinguish CU individuals from those with aMCI by capturing subtle impairments in IADLs and AADLs that are increasingly relevant in modern life. These findings highlight the importance of considering not only cognitive status but also the evolving functional demands driven by societal changes and the widespread integration of ICT worldwide.

This study presents several limitations. First, this study was conducted in a single urban setting–health centers in Callao, a city adjacent to Lima–where access to technology and healthcare is likely more developed than in other regions of Peru. As approximately 30% of Peru’s population resides in Lima, this concentration may have introduced selection bias and limited the external validity of our findings, particularly regarding their applicability to rural or underserved populations. Moreover, Peru’s marked cultural diversity and regional disparities in education and ADL may influence both cognitive assessment and diagnostic outcomes. Therefore, results from Callao may not fully capture the realities of individuals living in less resourced or non-urban areas. Future studies should expand our work to include a larger sample size and multicenter studies from urban and rural contexts, representing a more diverse group (including speakers of native languages such as Quechua or Aymara) of patient–caregiver pairs. Additionally, no biological biomarkers of disease, such as cerebrospinal fluid biomarkers or amyloid PET, were collected, limiting the confirmation of underlying neuropathology (Dubois et al., 2024). Recent reports in the native Peruvian population suggest that plasma pTau-217 becomes more strongly associated with functional impairment as disease severity progresses. Notably, the PFAQ threshold of 10 may mark the transition from aMCI to early AD, where tau burden begins to manifest functionally (Brown et al., 2025). The cross-sectional design of our study captures a single snapshot in time, which limits inferences about the rate of functional decline, and longitudinal follow-up could also help determine whether T-ADLQ is an effective measure to monitor disease progression. Longitudinal studies are needed to confirm the trajectory of functional losses in aMCI and AD. A prospective follow-up would likely reveal even more pronounced divergence in how quickly everyday abilities deteriorate. Following patients over time could also establish whether specific early AADL and IADL deficits predict subsequent cognitive decline or conversion from aMCI to AD.

Finally, future research should aim to validate the T-ADLQ across different dementia etiologies–such as AD, frontotemporal dementia, and vascular dementia–as patterns of functional impairment may vary by subtype. It is also essential to examine how educational levels and other social determinants of health influence functional limitations, particularly in complex ADL involving technological or cognitively demanding tasks. This is especially relevant in populations with no formal education, where conventional tools may fail to detect early functional decline. Exploring specific ADL limitations could help clarify how schooling modulates functional performance, supporting the development of more inclusive assessment instruments (Verbrugge and Jette, 1994). Comparative studies, including control groups with formal education, would further enhance understanding of the contextual validity and applicability of these tools across diverse clinical and sociocultural settings.

In conclusion, the total T-ADLQ scale demonstrated strong psychometric properties, including high validity, reliability, and discriminative capacity. These findings support its utility as a tool for evaluating functional impairment in individuals with ADD and for distinguishing them from CU and aMCI groups, including those with no formal education. Clinically, this underscores the importance of developing and validating functional assessment instruments that are appropriate for populations with diverse educational and cultural backgrounds. From a public health perspective, the capacity to detect early functional changes in underserved or low-literacy populations is critical for timely diagnosis, care planning, and allocation of resources. These results further emphasize the need for inclusive assessment tools to inform equitable health policies that address the needs of aging populations in diverse sociocultural settings.

## Data Availability

The raw data supporting the conclusions of this article will be made available by the authors, without undue reservation.
